# Supplementary material for: Content-rich biological network constructed by mining PubMed abstracts
Source: BMC Bioinformatics. 2004 Oct 8;5:147. doi: 10.1186/1471-2105-5-147 (PMC528731; doi:10.1186/1471-2105-5-147)
Supplement: Additional File 2 — The original results of the above study (non-essential files are deleted to keep the file size under the limit set by BMC bioinformatics). [file 1471-2105-5-147-S2.bz2 › chilibotAdditionalFile2/dip05/58ID8414517E213/html/RBBP1_RB1.html]

 


 **RBBP1** and **RB1** 
  
Found 17 abstracts in PubMed, retrieved 05.  
 

 What does Google say? 
 PDF only 
| .edu only 

---

**Interactive relationship** (e.g. stimulation, inhibition, etc)

**Inhibitory relationship**- RBP1  [ **RBBP1** ]  recruits the mSIN3 histone deacetylase complex to the pocket of retinoblastoma  [ **RB1** ]  tumor suppressor family proteins found in limited discrete regions of the nucleus at growth arrest.  Ref: 11283269 Mol Cell Biol, 2001
**Neutral relationship**

**Non-interactive relationship** (e.g. studied together, co-existance, homology, etc.)

- Clustering was successfully completed for DDP, resulting in selection of 23 candidates as DDP resistance related genes, including vascular permeability factor, 2 membrane transporting subunits, and retinoblastoma  [ **RB1** ]  binding protein 1  [ **RBBP1** ] .  Ref: 12824904 Cancer Sci, 2003
- In this report, we used an immunoglobulin G IgG purified from serum of a patient with breast cancer to identify its corresponding epitope, gene, and protein retinoblastoma  [ **RB1** ]  binding protein 1  [ **RBBP1** ]  like protein 1 RBP1L1 and determined whether it is a potential molecular marker for various cancers.  Ref: 11481388 J Natl Cancer Inst, 2001
- Five days later, the cultures were analyzed for cell survival, activation, DNA synthesis, and expression of EBV encoded and of cellular regulatory proteins retinoblastoma  [ **RB1** ]  Rb, p53, recombinant sequence binding protein RBP  [ **RBBP1** ]  Jk, and PU.1.  Ref: 10881992 J Hum Virol< MedlineTA>J Hum Virol, 2001
- The derived protein sequence of RBP1L1 shares 74% and 37% amino acid identity, respectively, with a partial sequence of the retinoblastoma  [ **RB1** ]  binding protein and the complete sequence of retinoblastoma  [ **RB1** ]  binding protein 1  [ **RBBP1** ] .  Ref: 11481388 J Natl Cancer Inst, 2001
- Initial computer based similarity searches identified human retinoblastoma  [ **RB1** ]  binding protein 1  [ **RBBP1** ]  RBP 1  [ **RBBP1** ] , Drosophila melanogaster male specific lethal 3 Msl 3, S. pombe altered polarity 13 Alp13 and S. cerevisiae Eaf3p, a component of the yeast NuA4 HAT complex Galarneau et al.  Ref: 11290425 Gene, 2001
